# Benchmarking Triage Capability of Symptom Checkers Against That of Medical Laypersons: Survey Study

**DOI:** 10.2196/24475

**Published:** 2021-03-10

**Authors:** Malte L Schmieding, Rudolf Mörgeli, Maike A L Schmieding, Markus A Feufel, Felix Balzer

**Affiliations:** 1 Department of Anesthesiology and Operative Intensive Care Charité – Universitätsmedizin Berlin, corporate member of Freie Universität Berlin and Humboldt-Universität zu Berlin Berlin Germany; 2 Institute of Medical Informatics Charité – Universitätsmedizin Berlin, corporate member of Freie Universität Berlin and Humboldt-Universität zu Berlin Berlin Germany; 3 Department of Biology, Chemistry, and Pharmacy Institute of Pharmacy Freie Universität Berlin Berlin Germany; 4 Department of Psychology and Ergonomics (IPA) Division of Ergonomics Technische Universität Berlin Berlin Germany

**Keywords:** digital health, triage, symptom checker, patient-centered care, eHealth apps, mobile phone, decision support systems, clinical, consumer health information, health literacy

## Abstract

**Background:**

Symptom checkers (SCs) are tools developed to provide clinical decision support to laypersons. Apart from suggesting probable diagnoses, they commonly advise when users should seek care (*triage advice*). SCs have become increasingly popular despite prior studies rating their performance as mediocre. To date, it is unclear whether SCs can triage better than those who might choose to use them.

**Objective:**

This study aims to compare triage accuracy between SCs and their potential users (ie, laypersons).

**Methods:**

On Amazon Mechanical Turk, we recruited 91 adults from the United States who had no professional medical background. In a web-based survey, the participants evaluated 45 fictitious clinical case vignettes. Data for 15 SCs that had processed the same vignettes were obtained from a previous study. As main outcome measures, we assessed the accuracy of the triage assessments made by participants and SCs for each of the three triage levels (ie, *emergency care*, *nonemergency care*, *self-care)* and overall, the proportion of participants outperforming each SC in terms of accuracy, and the risk aversion of participants and SCs by comparing the proportion of cases that were overtriaged.

**Results:**

The mean overall triage accuracy was similar for participants (60.9%, SD 6.8%; 95% CI 59.5%-62.3%) and SCs (58%, SD 12.8%). Most participants outperformed all but 5 SCs. On average, SCs more reliably detected emergencies (80.6%, SD 17.9%) than laypersons did (67.5%, SD 16.4%; 95% CI 64.1%-70.8%). Although both SCs and participants struggled with cases requiring self-care (the least urgent triage category), SCs more often wrongly classified these cases as emergencies (43/174, 24.7%) compared with laypersons (56/1365, 4.10%).

**Conclusions:**

Most SCs had no greater triage capability than an average layperson, although the triage accuracy of the five best SCs was superior to the accuracy of most participants. SCs might improve early detection of emergencies but might also needlessly increase resource utilization in health care. Laypersons sometimes require support in deciding when to rely on self-care but it is in that very situation where SCs perform the worst. Further research is needed to determine how to best combine the strengths of humans and SCs.

## Introduction

### Use of Symptom Checkers

Patients obtain health-related information from health care professionals, but more frequently, information for patients is provided in print; on the web; and, most recently, via smartphone apps. Patients not only use these resources to supplement information received from health care professionals but also as a decision-support tool to advise them on whether and where to seek adequate health care, especially as health care pathways grow more complex. Symptom checkers (SCs) are tools developed to provide support to laypersons. Users can enter their complaints and, with some SCs, demographic or health-related information (eg, age, sex, and past medical history) to obtain advice on the urgency of their complaints (*triage advice*) and the most likely diagnosis. The demand for this type of support is evident; in the United States, 1 in 3 people reported resorting to the internet for self-diagnosis [1], and a study from 2019 found that half of the patients involved in that study had investigated their symptoms with an online search engine before going to an emergency department [2].

### Evidence on SCs

Despite their popularity, there is no established framework to evaluate the performance of SCs [3,4]. The use of case vignettes, based on real or fictitious patients, has been a common approach for rating SCs [5-9]. The 2 most recent non–industry-funded audit studies using this methodology rated SC triage capability as unreliable, with an average of only 49% and 58% of appraisals deemed correct [10,11]. In line with these findings, a 2020 literature review concluded that most investigated SCs offered limited benefits [12].

A study showing that laypersons are just as capable of predicting criminal recidivism as a complex commercial algorithm [13] inspired us to compare the triage capability of SCs with that of participants with little or no medical training: are SCs merely a more complicated means of pointing out what an untrained individual could just as easily deduce? Is there an advantage to consulting SCs instead of relying on one’s own judgment?

In addition to advising the individual user, SCs are also said to have the potential to reduce the burden on health care services. Unfortunately, not only has this potential benefit not materialized yet [3] but also there is evidence of the opposite effect, as overly risk-averse SCs promote more visits to emergency care services [14]. To address this issue, we also analyzed whether SCs were more risk averse than our participants. Although SCs can also provide diagnostic suggestions, we considered triage advice to be more relevant for assessing the impact of SC on use of health care resources and patient safety.

The purpose of this study is to benchmark the triage capability of SCs against that of their potential users, that is, laypersons.

## Methods

### Ethics Approval and Consent to Participate

This study was approved by the Ethics Committee of the Department of Psychology and Ergonomics (Institut für Psychologie und Arbeitswissenschaft) at Technische Universität Berlin (tracking number: FEU_03_20180615). Participants volunteered to participate in the survey, and informed consent was required.

### Data Collection

This investigation builds on a prior study by Semigran et al [11], who evaluated SC triage performance based on case vignettes. We used their results on the performance of SCs as well as their case vignettes. Data were collected to determine the triage ability of medical laypersons, which was then used as a benchmark for comparing laypersons’ performance with that of SCs.

### Participants

All participants were US residents, at least 18 years of age, and had no professional medical background. Our investigation was limited to US residents, as the triage level definitions and the gold standard solutions assigned to the case vignettes by Semigran et al [11] might only be applicable to the US health care environment and might not apply to other health care systems with different service provider options.

### Survey

We created an online survey with UNIPARK (QuestBack GmbH) [15] containing questions on demographics (age, sex, US residency, and highest level of completed formal education), past online searching behavior for medical information, 45 randomly ordered clinical case vignettes, and 5 attention checks (see *Procedure* for further details). We used the 45 case vignettes compiled and adjusted by Semigran et al [11], which are between 1 and 3 sentences long and describe a patient’s signs and symptoms and occasionally mention elements of the patient’s past medical history.

Participants were asked to classify each vignette into 1 of 3 triage categories, as defined by Semigran et al [11]: *emergency care*, involving “the advice to call an ambulance, go to an emergency department, or see a general practitioner immediately”; *nonemergency care*, which encompasses “advice to call a general practitioner or primary care provider, see a general practitioner or primary care provider, go to an urgent care facility, go to a specialist, go to a retail clinic, or have an e-visit”; and *self-care*, which is “advice to stay at home or go to a pharmacy.” The definition of each triage level was explained at the beginning of the survey. The understanding of these definitions by participants was ascertained by 3 control questions given before the case vignettes were presented. The questionnaire was piloted with 12 participants and refined according to their feedback to ensure readability and understandability.

### Preparing the Case Vignettes

The 45 standardized case vignettes included 15 cases for each triage level. The vignettes, as chosen by Semigran et al [11], included both common and uncommon conditions with a wide range of chief complaints. The vignettes stemmed from various clinical sources, including material used to educate health care professionals.

For the purpose of our study, the vignettes were adapted to increase the comprehensibility of lay individuals. First, we transformed the bullet points into complete sentences. Second, we paraphrased technical terms. For example, we replaced “rhinorrhea” with “runny nose” and “tender” with “painful to the touch.” In very few cases, explanations required elaboration. Our overall aim was to provide participants with the same information used by Semigran et al [11] to assess SCs. We deemed 1 case vignette vague regarding a crucial piece of information and had to supplement it with a detail left out in the Semigran et al [11] version of the vignette (see Multimedia Appendix 1 [11] for details). We retained the classification of the 45 case vignettes into 3 triage levels.

Understandability and paraphrasing were cross-validated by two native English speakers: one was a medical professional (RM) and the other was without a professional medical background (MALS). The adapted vignettes are shown in Multimedia Appendix 1.

### Procedure

We recruited the participants through Amazon Web Service *Amazon Mechanical Turk* (MTurk), as it provides an established means to recruit US-based participants for sociopsychological surveys and is easy to access for researchers working outside of the United States [16]. Each participant received US $4.00 for completing the survey and a US $3.00 bonus if their overall accuracy in assigning the correct triage level was greater than or equal to 58%. The bonus was intended to provide an incentive for participants to pay close attention to the case vignettes and to assess a case’s urgency as accurately as possible. The chosen threshold of 58% corresponds to outperforming the SC average reported by Semigran et al [11].

Two methods were employed to ensure that the participants paid close attention to the survey questions. First, we added 5 attention checks to the set of 45 case vignettes. These attention checks were formatted similarly to the case vignettes but included prompts to choose specific answer options. Participants were excluded from the analysis if they answered any of the 5 attention checks incorrectly. Second, upon completion of the survey, participants were asked to affirm that they were attentive and honest to improve the reliability of our data, as suggested in a reliability analysis on MTurk data [17]. We assured participants that they would be compensated for completing the survey even if they stated that they had responded inattentively or dishonestly. We analyzed data only from participants who affirmed their honesty and attentiveness.

The survey on MTurk was published on 3 different days (March 21, 2020, at 2 PM Pacific Daylight Time [PDT]; March 22, 2020, at 1:45 PM PDT; and March 29, 2020, at 1 PM PDT). By selecting the weekend day and early afternoon PDTs, we attempted to reach an MTurk population as diverse as possible, following a 2017 study on the intertemporal variation of the MTurk population [18]. On each day, participants were recruited within a few hours of publishing the survey.

Due to limited funding, the sample size was ultimately determined by the availability of funds and the number of participants who performed well enough to earn a bonus.

### Data Analysis

Data were cleaned and explored using *R* 4.0.0 [19] and *tidyverse* packages [20]. Inferential analysis was conducted using the packages *lme4* [21] and *infer* [22]. Figures were created using the package *ggplot2* [23]. The data set containing participants’ triage assessments and their demographic variables was made publicly available [24].

Following Semigran et al [11], we refer to each instance of an SC or a participant assessing a case vignette as a “case evaluation.” For example, 2 participants each assessing all 45 case vignettes yielded 90 case evaluations.

#### Participant Characteristics

To assess the effects of demographic variables (age, sex, and educational level), a logistic regression was performed with the correct triage of a case vignette as a dependent variable. We calculated 95% CIs for the marginal probabilities of the fixed effects using the Wald method to assess whether demographic variables had a significant effect on participants’ accuracy. The α level was set at .05.

#### Comparing SCs and Participants

For the comparison of SCs and participants, we performed (1) a comparison between participants and all rated SCs aggregated and (2) between participants and individual SCs.

##### Aggregate Comparison of SCs and Participants

The performance of the SCs was obtained from the appendix of the audit study by Semigran et al [11]. Comparisons were made between SCs and participants in terms of (1) triage accuracy, (2) tendency to overtriage (*risk aversion*), and (3) how difficult each case vignette was for the respective group (SCs and participants). Of the 15 SCs, 4 (*iTriage*, *Isabel*, *Symcat*, and *Symptomate*) were designed to never suggest self-care, with 1 SC (*iTriage*) always advising users to seek emergency care. To ensure that our results were not skewed by these special SCs, we conducted the main aggregate analyses twice, including and excluding those 4 SCs, and reporting results for both.

###### Triage Accuracy

Following Semigran et al [11], we compared the performance of SCs and participants at an aggregate level and for each triage level separately and overall. This was performed by calculating the sample’s mean accuracy for SCs and participants, with accuracy defined as the proportion of vignettes solved correctly. For the participants, the standard error of the sampling mean with 95% CIs was estimated by bootstrapping the participant data with 15,000 replications. The limits of the CI were calculated using the quantile method (2.5th and 97.5th quantile of the bootstrap sample means). The CIs for the SC sample were not calculated, as Semigran et al [11] sampled the SCs purposefully, that is, they selected which SCs to evaluate with care and not randomly.

###### Risk Aversion

The risk aversion of the SCs and the participants was determined using the ratio of overtriaged vignettes to undertriaged vignettes. We deemed a ratio greater than 1:1, which is more case vignettes overtriaged than undertriaged, as risk averse. To determine what type of triage mistakes were most likely to occur, we calculated the proportion of triage recommendations given in each triage category by SCs and by participants (eg, the proportion of evaluations in which participants recommended emergency care when self-care was appropriate or the proportion of evaluations in which SCs recommended nonemergency care when emergency care would have been the correct solution) and compared these proportions using the Pearson *χ*² test.

###### Difficulty of Case Vignettes

To analyze whether SCs and participants were challenged by the same case vignettes, the degree of difficulty of a case was calculated using the proportion of SCs and participants correctly triaging it. For example, if a case vignette was solved correctly by every SC, the vignette’s degree of difficulty for SCs was 100%. SCs that did not evaluate the respective case vignette for technical reasons were not included in the denominator. A linear correlation analysis was then conducted to determine the relationship between case difficulty for SCs and case difficulty for participants.

##### Comparing Individual SCs With Participants

As users are likely to use only one or very few SCs, there is no basis for recommendations about using or not using SCs on an aggregated analysis alone. Therefore, additional analyses compared the performance of the participant group with each SC. Considering that most SCs did not evaluate every case vignette (due to technical reasons, see the study by Semigran et al [11]), the triage accuracy of the participants was calculated using only the cases evaluated by a specific SC, enabling a direct comparison. The CIs for participants’ mean accuracy were calculated as described above. We also determined the proportion of participants that managed to achieve higher accuracy across cases than the respective SC. Furthermore, risk aversion was also evaluated, given the specific set of case vignettes for any given SC by plotting the proportion of vignettes that were overtriaged against the proportion of those undertriaged for participants versus SC.

## Results

### Participant Characteristics

Our survey was accessed 142 times in 3 days during which it was available in total, 51 participants were excluded, either for failing attention checks (n=41) or for not fulfilling the eligibility criteria (n=10). All the remaining participants affirmed that they had paid close attention during the survey and answered honestly. This yielded a total of 91 participants, each having assessed all 45 case vignettes, which totaled 4095 case evaluations by participants, 1365 for each triage level (Table 1).

The median time for completion of the survey (excluding the time for obtaining informed consent) was 20 minutes and 12 seconds (1st quartile=15 minutes:43 seconds; 3rd quartile=27 minutes:23 seconds). There was no significant difference in the participants’ mean accuracy between the 3 sampling days. We detected no statistically significant influence of demographic variables on participants’ triage accuracy.

**Table 1:** Participant characteristics (N=91).

Characteristics		Values
Age (years), median (range)		37 (20-73)
**Gender, n (%)**		
	Female	36 (40)
	Male	55 (60)
**Education, n (%)**		
	Non–high school graduate	0 (0)
	High school graduate	18 (20)
	Some college	33 (36)
	Bachelor’s degree	36 (40)
	Graduate degree	4 (4)
**Recent^a^ triage experience, n (%)**		
	Recently consulted an SC	20 (22)
	Recently faced triage decision	23 (25)
	Neither faced triage decision nor consulted an SC recently	62 (69)
**Medical training, n (%)**		
	No training	80 (88)
	Basic first aid training	11 (12)

^a^Recent was defined as “in the last 6 months.”

### Comparing SCs’ and Participants’ Triage Performance

#### Participant Performance

Overall, the participants triaged 3 out of 5 case vignettes correctly (2462/4065, 60.57%), and most participants qualified for the bonus payment (56/91, 62%). Their mean accuracy varied with triage level, roughly balanced for emergency and nonemergency situations (67.5% and 68.4%, respectively) but dropped below 50% for self-care vignettes. Of the 39.43% (1603/4065) of incorrect assessments, the majority (956/4065, 23.52%) were *overtriaged,* that is, participants assigned a more urgent triage level than necessary. Only about every sixth case vignette was *undertriaged* (647/4065, 15.92%), that is, participants assigned a less urgent triage level than necessary.

#### Aggregated Comparison Analyses

As most SCs were unable to evaluate at least one of the case vignettes, the 15 SCs assessing the 45 case vignettes yielded only 532 case evaluations (see the study by Semigran et al [11] for details): 183 for emergency vignettes, 175 for nonemergency vignettes, and 174 for self-care vignettes.

##### Triage Accuracy

At the aggregate level, SCs (58.0%; SD 12.8%) and participants (60.9%; SD 6.8%) showed very similar mean accuracies (Table 2). This remains to be the case when excluding the 4 SCs that did not suggest self-care (adjusted mean for the 11 SCs; 61.6%; SD 11.0%). Table 2 shows that differences become apparent when evaluating the triage levels separately: for emergency case vignettes, SCs outperformed the participants, whereas the participants outperformed the average SC in the nonemergency and self-care cases. For the least urgent triage level, this difference decreases when excluding those SCs that never recommend self-care.

**Table 2:** Mean triage accuracy of symptom checkers and participants.

Triage level	Percent triage accuracy, mean (SD)			95% CI
	All 15 SCs^a^	Subset of 11 SCs^b^	Participants^c^	
Emergency cases	80.6 (17.9)	79.8 (17.2)	67.5 (16.4)	64.1-70.8
Nonemergency cases	58.5 (29.1)	61.6 (27.8)	68.4 (13.8)	65.6-71.2
Self-care cases	30.6 (25.7)	41.8 (20.3)	46.7 (15.9)	43.4-49.8
Overall	58.0 (12.8)	61.6 (11.0)	60.9 (6.8)	59.5-62.3

^a^SC: symptom checker.

^b^For the subset of 11 SCs, SCs never recommending self-care or always recommending emergency care by design were excluded.

^c^For the participant sample, 95% CIs were calculated using bootstrapping.

##### Risk Aversion

**Figure 1:**
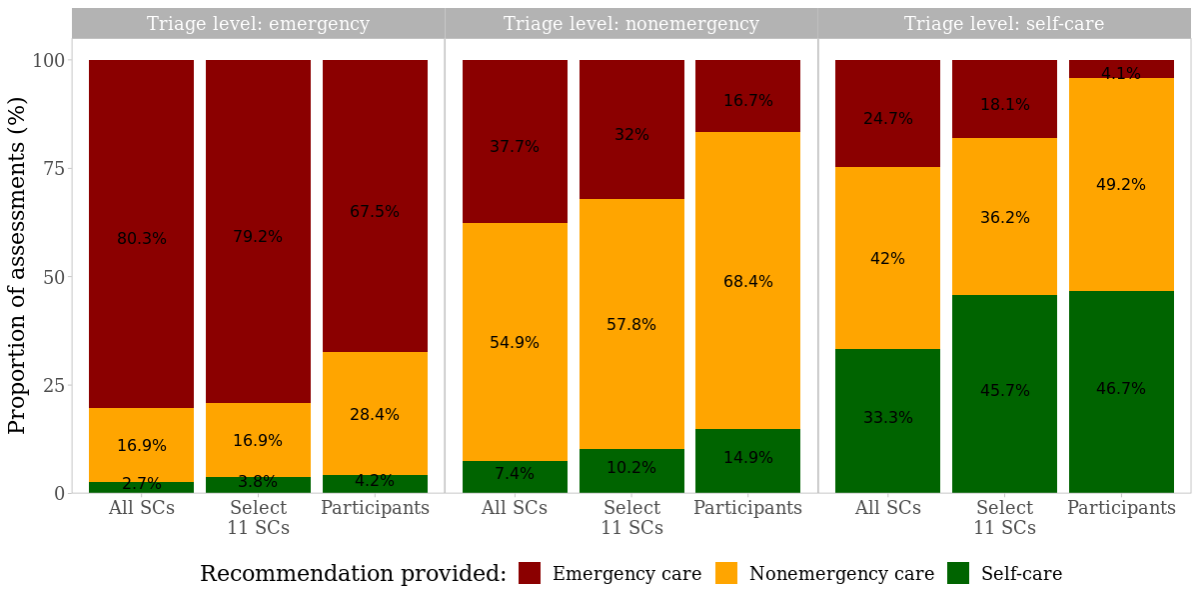
Triage evaluations by participants and SCs and triage level. “11 SCs” refers to the SC sample after exclusion of SCs that never recommend self-care (the least urgent triage level). SC: symptom checker.

The SCs were risk averse and overtriaged in more than a third of the evaluations (182/532, 34.2%), whereas undertriaging occurred in only 9.2% (49/532). Although participants also tended to be risk averse, this tendency was less pronounced (Figure 1). The ratio of overtriage to undertriage errors was 1.5:1 for participants whereas it was 3.5:1 for SCs. The SCs misclassified self-care cases as emergencies 6 times more often than participants did (43/174, 24.7% vs 56/1365, 4.10%) and 4.5 times more often (23/127, 18.1% vs 56/1365, 4.1%) when considering the subset of 11 SCs. The pair-wise differences in recommendations per triage level were statistically significant between participants and SCs (*P*=.002 for triage-level emergency [*χ*²_2_=12.5]; *P*<.001 for nonemergency [*χ*²_2_=46.3] and self-care [*χ*²_2_=109.6]). This holds true when comparing the participants’ performance with the subset of 11 SCs (*P*=.02 for an emergency [*χ*²_2_=8.1] and *P*<.001 for a nonemergency [*χ*²_2_=19.0] and for self-care [*χ*²_2_=47.1]).

##### Comparing Case Vignette Difficulty for SCs and for Participants

**Figure 2:**
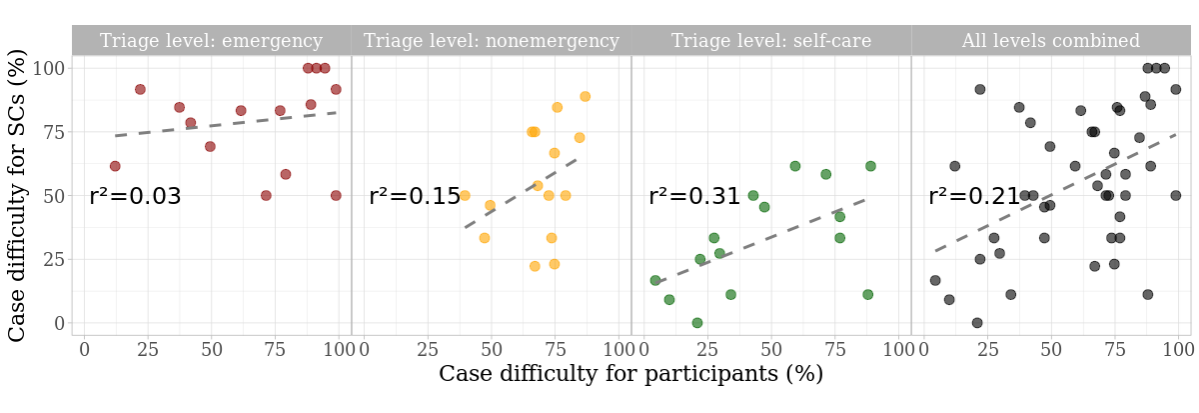
Distribution of case difficulty for participants and SCs. Case difficulty is defined as the proportion of the group (SC or participants) evaluating the respective case correctly. The dashed line models a linear relationship. SC: symptom checker.

How challenging a case vignette was for SCs and participants varied widely: 3 vignettes were solved correctly by every SC and 1 vignette by none. Similarly, 4 vignettes were solved correctly by more than 90% of the participants and 2 by less than 10%. At every triage level, a broad variation in the degree of difficulty among case vignettes was observed. A very weak or no relationship could be detected for SCs and participants regarding case difficulty within each triage level (Figure 2).

##### Comparing Individual SCs With Participants

**Figure 3:**
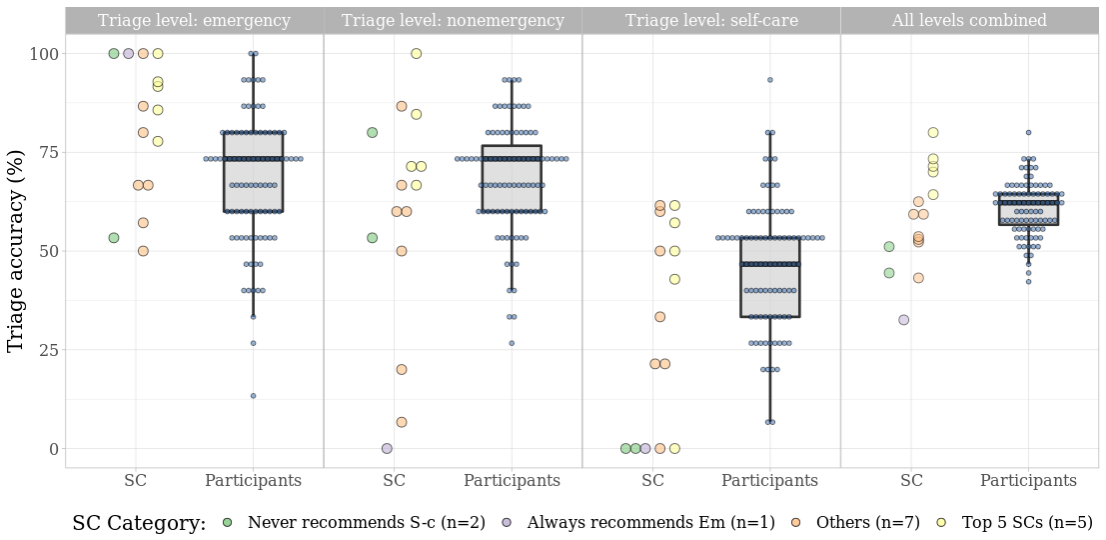
Accuracy of SCs and participants by triage level (Em), nonemergency, and S-c. The accuracy of individual participants is indicated with blue dots. The aggregate accuracies of participants are shown as box plots. Em: emergency; SC: symptom checker; S-c: self-care.

As previously mentioned, an aggregated analysis of SCs is less meaningful than a direct comparison between the participant population and each SC, as users are likely to consult only one or very few SCs. The overall trend shows that the accuracy of both participants and SCs decreases for self-care vignettes (Figure 3).

A total of 5 SCs (*HMS* [Harvard Medical School] *Family Health Guide*, *Healthy Children*, *Steps2Care*, *Symptify*, and *Symptomate*) managed to outperform the participant sample, achieving an overall accuracy greater than the mean of the participants and its CI’s upper limit (Table 3; see yellow dots in Figure 3). Five SCs had a triage capability lower than 80% (73/91) of the participants. This finding is partially explained by 3 of them apparently designed to never recommend self-care, hence failing in one-third of the cases owing to their design. One of these 3 SCs (*Isabel*) was outperformed only by a minority of participants (17/91, 18%), when self-care case vignettes were excluded from the analysis. The remaining 2 SCs (*Symcat* and *iTriage*) were still outperformed by most participants when self-care case vignettes were excluded. The participants’ mean accuracy was stable at approximately 60%, independent of the slightly different samples of vignettes assessed by the SCs, with 2 exceptions: the participants were challenged by the sample of vignettes evaluated by *Healthy Children*, reaching a mean accuracy that was approximately 10% lower than in the other samples; conversely, the participants fared much better in assessing the vignette sample considered by *DoctorDiagnose*.

**Figure 4:**
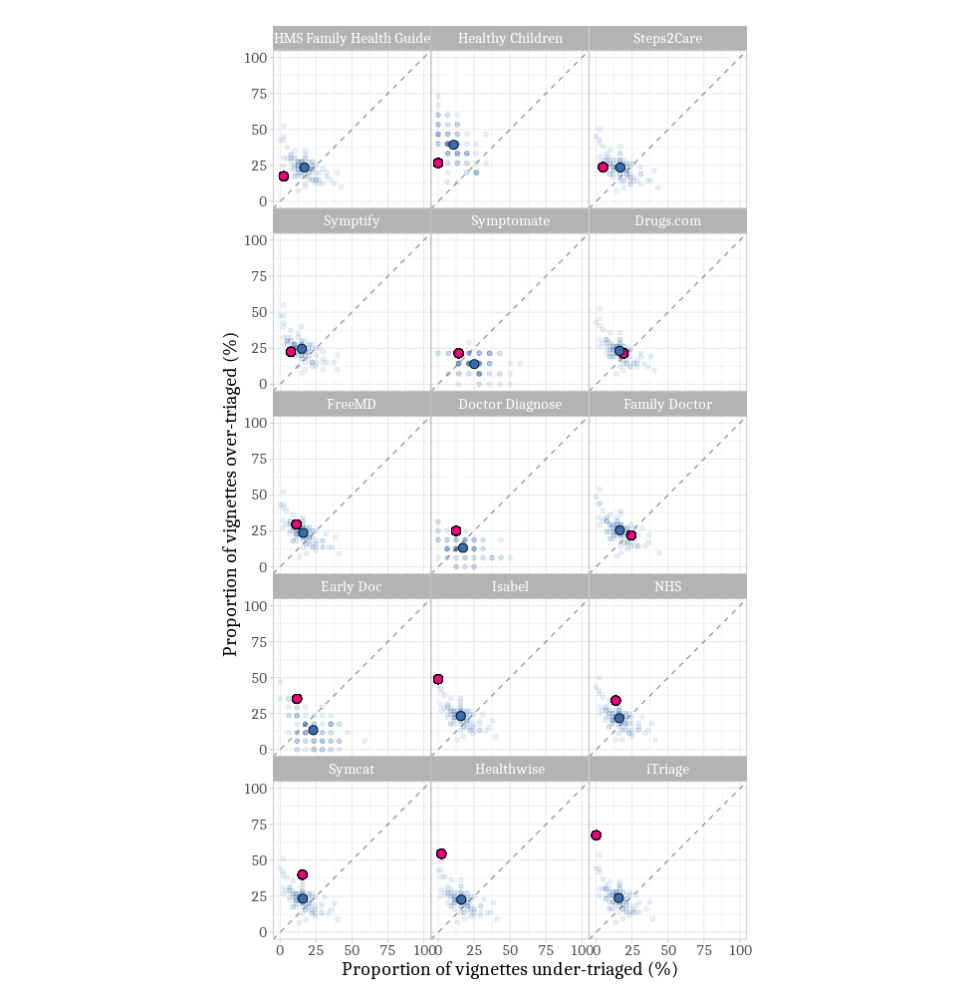
Comparison of the overtriage inclination of symptom checkers (SCs) and participants. The dashed line shows where proportions of over and undertriaged errors are equal. Proximity to the left lower corner indicates a high triage accuracy. The red dot marks the respective symptom checker. The faded blue dots refer to the performance of individual participants. The larger blue dot marks their average performance. The SCs are ordered from left to right and top to bottom by the proportion of participants outperforming them, with the lowest proportional difference at the top left and the highest proportional difference on the bottom right.

All but 2 SCs (*Family Doctor* and *Drugs.com*) were risk averse, making more overtriage errors than undertriage errors. Although the best 5 SCs were inclined toward overtriage, only one of them overtriaged more vignettes than the average participant (*Symptomate*; Figure 4).

**Table 3:** Comparison of accuracy between symptom checkers and participants.

SC^a,b^ name	Accuracy^c^, n (%)	Participants		Comparison
		Percent accuracy^d,e^, mean (SD)	95% CI	Percentage of participants outperforming the SC (95% CI)^d,e^
HMS^f^ Family Health Guide, n=40	32 (80)	59.5 (7.1)	58.0-60.9	0 (0-0)
Healthy Children, n=15	11 (73)	49.9 (10.1)	47.7-52.1	1.1 (0-3.3)
Steps2Care, n=42	30 (71)	59.7 (7.2)	58.2-61.1	1.1 (0-3.3)
Symptify, n=40	28 (70)	60.2 (7.2)	58.2-61.7	5.5 (1.1-11.0)
Symptomate^g^, n=14	9 (64)	60.9 (11.6)	58.6-63.2	26.4 (17.6-35.2)
Drugs.com, n=42	25 (59)	60.6 (6.5)	59.3-61.9	51.6 (41.8-61.5)
FreeMD, n=44	26 (59)	60.2 (6.7)	58.9-61.6	56.0 (45.1-65.9)
Doctor Diagnose, n=16	10 (62)	69.5 (10.9)	67.3-71.7	63.7 (53.8-73.6)
Family Doctor, n=41	22 (53)	58.1 (7.0)	56.7-59.6	68.1 (58.2-78.0)
Early Doc, n=17	9 (52)	63.4 (11.4)	61.1-65.7	76.9 (68.1-85.7)
Isabel^g^, n=45	23 (51)	60.9 (6.8)	59.4-62.2	89 (82.4-94.5)
NHS^h^, n=44	23 (52)	62.0 (6.9)	60.9-63.4	89 (82.4-94.5)
Symcat^g^, n=45	20 (44)	60.9 (6.8)	59.5-62.2	97.8 (94.5-100)
Healthwise, n=44	19 (43)	61.2 (7)	59.7-62.6	98.9 (96.7-100)
iTriage^h,i^, n=43	14 (32)	60.5 (6.9)	59.1-61.9	100 (100-100)

^a^SC: symptom checkers

^b^SCs are listed in order by the proportion of participants outperforming them.

^c^Most SCs did not evaluate every case vignette. Their accuracy is given as the proportion of correctly solved vignettes of the total vignettes that they evaluated.

^d^The participants’ accuracy is based on their assessment of the same case vignettes assessed by the respective SC.

^e^For the participant sample, 95% CIs were calculated using bootstrapping.

^f^HMS: Harvard Medical School.

^g^Four SCs were apparently designed never to recommend self-care.

^h^NHS: National Health Service.

^i^One SC advised seeking emergency care for all case vignettes.

## Discussion

### Principal Findings

Our study suggests that an average SC has no greater overall triage accuracy than an average user. However, this does not imply that SCs are not useful. Specifically, our data confirm a prior study showing that the lay population has difficulties reliably identifying medical emergencies [25]. On average, participants failed to identify every third emergency, and 12% (11/91) of our participants identified emergencies less reliably than the worst-performing SC.

Most SCs tended to overtriage. From a clinical and legal perspective, it can make sense to accept the resulting inflated cost of false alarms to avoid potentially missing an emergency (*defensive decision making*). In contrast, false alarms raised by SCs can functionally exacerbate overcrowding in health care services. In fact, the ability of some SCs to reliably detect emergencies can be partially attributed to their general tendency—by design—to recommend emergency care even for self-care cases (the least urgent triage level) where no medical care is warranted. This trade-off must be considered before recommending their use.

Studies on the effects of SC advice on users are scarce. Therefore, general recommendations on whether laypersons should use SCs cannot be formulated as yet. On the basis of a detailed analysis of the performance variation among SCs and human decision makers, we showed that the five best SCs that Semigran et al [11] included in their sample outperformed almost all our participants and thus could be seen as beneficial to users. In contrast, SCs mistake self-care cases for emergencies a substantial number of times. This hints at SCs being better suited to help users who are looking for an answer on where they should seek professional help (ie, by discriminating between emergency and nonemergency cases) rather than on whether they should seek medical care at all (ie, by discriminating between self-care and non–self-care cases).

Finally, SCs and participants struggled with different kinds of case vignettes, that is, SCs performed poorly in some clinical situations, whereas in others, their performance was superior to that of their users. For example, the 15 pediatric cases evaluated by the SC *Healthy Children* appear to have been more challenging for participants (mean accuracy of 49.9%) than the 30 nonpediatric cases (mean accuracy of 66.3%). To provide a more differentiated picture of SC triage performance, further analyses should also investigate performance differences with respect to different types of cases.

### Limitations

Compared with the general population of the United States [26], our participants were better educated and included more men than women. The median and mean ages were similar to those of the general US population. One study suggests that the groups most likely to seek health information online are younger White females from high-income households, most with a bachelor’s degree or higher [1]. Most participants in a survey among users of a specific SC (Isabel) were female and White but older than the average population [27]. Despite the fact that our sample’s demographic distribution did not fully resemble the US population or, presumably, the population of SC users, we consider our findings to have at least some external validity for these populations, as demographic variables showed no significant influence on triage accuracy.

The data on SCs date back to a study published in 2015 [11], where the specific versions of the SCs assessed were not specified. Therefore, changes in performance due to possible upgrades were not considered. Such upgrades are likely, and new SCs have since entered the market. Other SCs included in the Semigran et al sample [11] are no longer available online, including the best-performing SC (*HMS Family Health Guide*). This speaks to the general problem that future research evaluating the performance of SCs will have to address the rapidly changing markets and technological developments.

As we built our study on the materials of the Semigran et al study [11], we also inherited their limitations: the chosen 45 case vignettes do not cover the entire spectrum of prehospital case presentations, especially with the omission of mental health–related scenarios. In addition, some case vignettes lacked a proper diagnosis and stated only the presenting complaints (eg, “Vomiting” for vignette 45, “Constipation” for vignette 40, “Back pain” for vignette 20). This prevented a plausibility check of the gold standard triage level that should be assigned to each vignette.

In general, assessing triage capability with case vignettes has limited validity. This limitation is arguably greater for human participants than for SCs. Although SCs assess a case with a set algorithm and are therefore dependent only on input, contextual (social, emotional, etc) factors play a greater role in human decision making. In a real-life setting, humans might also notice and process more or less information than presented in a case vignette. In addition, reading “back pain” in a dry case vignette is surely a different matter than experiencing it. Thus, our results might be more valid for situations where SC users utilize the tool to triage someone other than themselves. Research shows that this is common practice, as up to 50% of online health information seekers do so on behalf of someone else [1].

### Conclusions

Prior publications have emphasized the need for a framework within which the safety and usefulness of SCs should be analyzed. Assessing the average performance of SCs, as has often been done, fosters few actionable recommendations. Given the high-performance variability among SCs, we consider benchmarking with case vignettes as a valuable first step in identifying the best SCs, which could then be tested extensively against relevant competitors.

Although comparing SCs’ triage capability against that of health care professionals is certainly useful [28], this approach implicitly asks whether the former could replace the latter, rather than assessing whether and under which circumstances a user should rely on an SC or refrain from using it. Similar to the common practice of testing a new medicine against a placebo, we suggest that SCs should be benchmarked against a realistic alternative, for example, an SC user relying on his own appraisal (stand-alone triage capability).

Following this approach, our study suggests that the lay population would benefit from some SCs to some extent. Although SCs detect emergencies more reliably than the average user, they are more risk averse than the general population and recommend emergency care more often than is actually necessary. This is a cause for concern, as it might unnecessarily increase the burden on already overwhelmed health care services. Thus, advice on when not to seek emergency care would be the most useful feature of SCs, but it is precisely in that situation that they performed the worst. Further research should investigate which user groups benefit the most from using SCs and whether it is possible to identify the characteristics of scenarios where laypersons are superior to SCs in assessing triage levels. The detailed analyses presented in this paper provide a first step toward a framework for comparatively assessing the respective weaknesses and strengths of both SCs and human decision makers to be able to determine when humans should rely on SCs rather than on their gut feeling and vice versa.

## Appendix

Multimedia Appendix 1 Adapted case vignettes and case difficulty level.

## Abbreviations

HMS: Harvard Medical School
MTurk: Mechanical Turk
PDT: Pacific Daylight Time
SC: symptom checker
