# Correction: Benchmarking Triage Capability of Symptom Checkers Against That of Medical Laypersons: Survey Study

**DOI:** 10.2196/30215

**Published:** 2021-05-06

**Authors:** Malte L Schmieding, Rudolf Mörgeli, Maike A L Schmieding, Markus A Feufel, Felix Balzer

**Affiliations:** 1 Department of Anesthesiology and Operative Intensive Care Charité – Universitätsmedizin Berlin, corporate member of Freie Universität Berlin and Humboldt-Universität zu Berlin Berlin Germany; 2 Institute of Medical Informatics Charité – Universitätsmedizin Berlin, corporate member of Freie Universität Berlin and Humboldt-Universität zu Berlin Berlin Germany; 3 Department of Biology, Chemistry, and Pharmacy Institute of Pharmacy Freie Universität Berlin Berlin Germany; 4 Department of Psychology and Ergonomics (IPA) Division of Ergonomics Technische Universität Berlin Berlin Germany

In “Benchmarking Triage Capability of Symptom Checkers Against That of Medical Laypersons: Survey Study” (J Med Internet Res 2021;23(3):e24475) the authors noted two errors.

In the originally published manuscript, Reference 24 in the reference list was cited incorrectly. This citation has now been corrected to the reference listed below [1].

In the originally published paper, there was an error in the phone number of the Corresponding Author. The number has been corrected from "49 30 450 5704" to "49 30 450 570425".

The correction will appear in the online version of the paper on the JMIR Publications website on May 6, 2021, together with the publication of this correction notice. Because this was made after submission to PubMed, PubMed Central, and other full-text repositories, the corrected article has also been resubmitted to those repositories.
