# Supplementary material for: Benchmarking Triage Capability of Symptom Checkers Against That of Medical Laypersons: Survey Study
Source: J Med Internet Res. 2021 Mar 10;23(3):e24475. doi: 10.2196/24475 (PMC7991983; doi:10.2196/24475)
Supplement: Multimedia Appendix 1 [file jmir_v23i3e24475_app1.docx]

**Supplementary Table 1: Adapted case vignettes and case difficulty level**

| **N°** | **Original version^[[1]](#endnote-1)^** | **Semigran’s abbreviated version^[[2]](#endnote-2)^** | **Vignette as used in our investigation** | **Commentary** | **Case difficulty level^[[3]](#endnote-3)^** |
| --- | --- | --- | --- | --- | --- |
| **Emergency cases** | | | | | |
| 1 | A 48-year-old woman with a history of migraine headaches presents to the emergency room with altered mental status over the last several hours. She was found by her husband, earlier in the day, to be acutely disoriented and increasingly somnolent. On physical examination, she has scleral icterus, mild right upper quadrant tenderness, and asterixis. Preliminary laboratory studies are notable for a serum ALT of 6498 units/L, total bilirubin of 5.6 mg/dL, and INR of 6.8. Her husband reports that she has consistently been taking pain medications and started taking additional 500 mg acetaminophen pills several days ago for lower back pain. Further history reveals a medication list with multiple acetaminophen-containing preparations. | 48 y/o f, confusion, disorientation, increasingly drowsy, mild right upper quadrant tenderness, chronic tylenol/acetaminophen - recently took more | “A 48-year-old woman is confused and disorientated. She feels increasingly drowsy, and the right side of her upper abdomen is painful to the touch. She has been taking a painkiller called Tylenol/acetaminophen (also known as “Paracetamol”) frequently, and has recently increased the dosage.” | Paracetamol explained | SCs: 50%  Subjects: 71.4% |
| 2 | A 12-year-old girl presents with sudden-onset severe generalized abdominal pain associated with nausea, vomiting, and diarrhea. On exam she appears ill and has a temperature of 104°F (40°C). Her abdomen is tense with generalized tenderness and guarding. No bowel sounds are present. | 12 y/o f, sudden onset severe abdominal pain, nausea, vomiting, diarrhea, T=104 | “A 12-year-old girl suddenly experiences severe stomach pain, with nausea and vomiting. She also has diarrhea and a fever of 104° Fahrenheit (40° Celsius).” |  | SCs: 100%  Subjects: 87.9% |
| 3 | A 27-year-old woman with a history of moderate persistent asthma presents to the emergency room with progressive worsening of shortness of breath, wheezing, and cough over 3 days. She reports prior exposure to a person who had a runny nose and a hacking cough. She did not receive significant relief from her rescue inhaler with worsening symptoms, despite increased use. She has been compliant with her maintenance asthma regimen, which consists of an inhaled corticosteroid and a leukotriene receptor antagonist for maintenance therapy and albuterol as rescue therapy. Her cough is disrupting her sleep pattern and as a consequence she is experiencing daytime somnolence, which is affecting her job performance. | 27 y/o f, Hx of asthma, mild shortness of breath, wheezing, 3 days cough, symptoms not responsive to inhalers, recent cold | “A 27-year-old woman with a history of asthma has shortness of breath and wheezing. She has been coughing for the past 3 days. Her symptoms do not improve when using her asthma inhaler. Recently she had a cold”. | In the original version the patient did not have a cold, but had contact to someone with a cold. To allow comparison between Semigran’s and our study’s data, we stuck to the Semigran version. | SCs: 91.6%  Subjects: 21.9% |
| 4 | A 67-year-old woman with a history of COPD presents with 3 days of worsening dyspnea and increased frequency of coughing. Her cough is now productive of green, purulent sputum. The patient has a 100-pack-year history of smoking. She has had intermittent, low-grade fever of 100°F (37.7°C) for the past 3 days and her appetite is poor. She has required increased use of rescue bronchodilator therapy in addition to her maintenance medications to control symptoms. | 67 y/o f, Hx of COPD, 3 days worsening shortness of breath, increase coughing, green sputum, low grade fever, increase use of rescue bronchodilator therapy | “A 67-year-old woman with a history of Chronic Obstructive Pulmonary Disease (“COPD”, a chronic lung disease) has shortness of breath, which has worsened over the last 3 days. She is coughing more often and is also now coughing up green mucus. She also has a low fever. She has to use her emergency inhaler (which opens up the airways to make breathing easier) more often to calm her symptoms”. | COPD explained | SCs: 69.2%  Subjects: 49.4% |
| 5 | A 65-year-old woman presents with unilateral leg pain and swelling of 5 days' duration. There is a history of hypertension, mild CHF, and recent hospitalization for pneumonia. She had been recuperating at home but on beginning to mobilize and walk, the right leg became painful, tender, and swollen. On examination, the right calf is 4 cm greater in circumference than the left when measured 10 cm below the tibial tuberosity. Superficial veins in the leg are more dilated on the right foot and the right leg is slightly redder than the left. There is some tenderness on palpation in the popliteal fossa behind the knee. | 65 y/o f, 5 days swelling, pain in one leg, recent hospitalization, leg painful, tender, swollen, red | “A 65-year-old woman has a swollen right leg. The leg has become painful in the past five days since leaving the hospital, and is now painful to the touch, now swollen and red.” | Clarified that only one leg is affected by adding a laterality (“right”) as in Semigran’s version the leg pain is mentioned twice. | SCs: 83.3%  Subjects: 61.5% |
| 6 | Mr. Y is a 64-year-old Chinese male who presents with chest pain for 24 hours. One day prior to presentation, the patient began to experience 8/10, non-radiating substernal chest pressure associated with diaphoresis and shortness of breath. The pain initially improved with Tylenol, however over the following 24 hours, his symptoms worsened. The patient went to his primary physician, where an EKG was performed which showed ST elevation in leads V2-V6. | 64 y/o m, 1-day chest pain (8/10), non-radiating substernal chest pressure, sweating, shortness of breath, (chest tightness) | “A 64-year-old man has been suffering from severe [8 on a scale of 0 to 10] chest pain for one day. He describes the pain as a pressure, which is localized under his breastbone (“chest tightness”). He is sweating and has trouble breathing.” |  | SCs: 100%  Subjects: 94% |
| 7 | A 4-year-old boy presents with a 7-day history of abdominal pain and watery diarrhea that became bloody after the first day. Three days before the onset of symptoms, he had visited the county fair with his family and had eaten a hamburger. Physical examination reveals a mild anemia | 4 y/o m, 7 day Hx of abdominal pain, bloody diarrhea, ate hamburger at fair 3 days ago | “A 4-year old boy with abdominal pain for 7 days has bloody diarrhea. Three days before the symptoms began, he ate a hamburger at a fair”. | Corrected Semigran’s vignette, as otherwise the case description would miss a crucial point: the eating the hamburger came three days before onset of symptoms not before presentation (“now”). | SCs: 83.3%  Subjects: 76.9% |
| 8 | A 45-year-old white man presents to the emergency department with a 1-hour history of sudden onset of left-sided flank pain radiating down toward his groin. The patient is writhing in pain, which is unrelieved by position. He also complains of nausea and vomiting. | 45 y/o m, 1 hour severe left-sided flank pain radiating into groin, nausea, vomiting, pain unrelieved by position | “A 45-year-old man developed severe pain on his left flank over the past hour. The pain radiates into his groin. He also has nausea and vomiting. The pain is not relieved with any change of position.” |  | SCs: 85.7%  Subjects: 89.0% |
| 9 | A 28-year-old man presents to his physician with a 5-day history of fever, chills, and rigors, not improving with acetaminophen (paracetamol), along with diarrhea. He had been traveling in Central America for 3 months, returning 8 weeks ago. He had been bitten by mosquitoes on multiple occasions, and although he initially took malaria prophylaxis, he discontinued it due to mild nausea. He does not know the specifics of his prophylactic therapy. On examination he has a temperature of 100.4°F (38°C), and is mildly tachycardic with a BP of 126/82 mmHg. The remainder of the examination is normal. | 28 y/o m, 5 day Hx of fever, chills, rigors, diarrhea, recent travel abroad to area with malaria, bitten by mosquitoes, did not take malaria prophylaxis consistently | A 28-year-old man has had a fever for five consecutive days. He has chills (i.e. has been shivering) and has diarrhea. He recently returned from an area abroad where malaria is present. Abroad, he was bitten by mosquitoes and did not consistently take medication for malaria prevention.” |  | SCs: 58.3%  Subjects: 79.1% |
| 10 | An 18-year-old male student presents with severe headache and fever that he has had for 3 days. Examination reveals fever, photophobia, and neck stiffness. | 18 y/o m, 3 days severe headache, fever, photophobia, neck stiffness | “An 18-year-old man has had a severe headache for 3 days. He is also suffering from fever, a stiff neck, and light-sensitivity." |  | SCs: 78.5%  Subjects: 41.7% |
| 11 | A 65-year-old man with hypertension and degenerative joint disease presents to the emergency department with a three-day history of a productive cough and fever. He has a temperature of 38.3°C (101°F), a blood pressure of 144/92 mm Hg, a respiratory rate of 22 breaths per minute, a heart rate of 90 beats per minute, and oxygen saturation of 92 percent while breathing room air. Physical examination reveals only crackles and egophony in the right lower lung field. The white-cell count is 14,000 per cubic millimeter, and the results of routine chemical tests are normal. A chest radiograph shows an infiltrate in the right lower lobe. | 65 y/o m, Hx of hypertension and degenerative joint disease, 3 day Hx of productive cough and fever (101) | “A 65-year-old man with a history of high blood pressure and a chronic joint disease has coughed up mucus for three days. He also has a fever of 101° Fahrenheit (38.3° Celsius)”. | Replaced “degenerative” with “chronic” to make the it more understandable to our lay participants | SCs: 61.5%  Subjects: 12.0% |
| 12 | A 65-year-old man presents to the emergency department with acute onset of SOB of 30 minutes' duration. Initially, he felt faint but did not lose consciousness. He is complaining of left-sided chest pain that worsens on deep inspiration. He has no history of cardiopulmonary disease. A week ago he underwent a total left hip replacement and, following discharge, was on bed rest for 3 days due to poorly controlled pain. He subsequently noticed swelling in his left calf, which is tender on examination. His current vital signs reveal a fever of 100.4°F (38.0°C), heart rate 112 bpm, BP 95/65, and an O2 saturation on room air of 91%. | 65 y/o m, shortness of breath for 30 min, chest pain that worsens with inspiration, recent surgery, recent bed rest, swelling in left calf, which is tender, fever | “A 65-year-old man has shortness of breath for 30 minutes and chest pain, which worsens when breathing in. He recently had a surgery, followed by a prolonged bed rest period. He also has a swollen left calf, which is painful to the touch, and fever”. | Added “prolonged” to bed rest. | SCs: 100%  Subjects: 91% |
| 13 | An 8-year-old boy in Oklahoma is brought to the emergency department over the fourth of July weekend because of fever, chills, malaise, arthralgias, and a headache. Physical examination reveals a maculopapular rash that is most prominent on his wrists and ankles. | 8 y/o m, Fever, chills, joint pain, headache, rash wrists/ankles | “A 8-year-old boy has fever, chills (i.e. has been shivering), joint pain, and a headache. He also has a rash over his wrists and ankles.” |  | SCs: 84.6%  Subjects: 37.3% |
| 14 | A 70-year-old man with a history of chronic HTN and atrial fibrillation is witnessed by a family member to have nausea, vomiting, and right-sided weakness, as well as difficulty speaking and comprehending language. The symptoms started with only mild slurred speech before progressing over several minutes to severe aphasia and right arm paralysis. The patient is taking warfarin. | 70 y/o m, nausea, vomiting, right-sided weakness, rt arm paralysis, difficulty speaking and comprehension | “A 70-year-old man suddenly develops nausea and vomiting. At first, he had weakness on the right side of his body and had trouble articulating words. The symptoms are worsening; now he is unable to move his right arm is unable to speak or understand words.” | Semigran version omitted atrial fibrillation which is presumably the cause of the stroke. We stuck with their version. | SCs: 91.6%  Subjects: 98.9% |
| 15 | A 63-year-old man sustained a cut on his hand while gardening. His immunization history is significant for not having received a complete tetanus immunization schedule. He presents with signs of generalized tetanus with trismus ("lock jaw"), which results in a grimace described as "risus sardonicus" (sardonic smile). Intermittent tonic contraction of his skeletal muscles causes intensely painful spasms, which last for minutes, during which he retains consciousness. The spasms are triggered by external (noise, light, drafts, physical contact) or internal stimuli, and as a result he is at the risk of sustaining fractures or developing rhabdomyolysis. The tetanic spasms also produce opisthotonus, board-like abdominal wall rigidity, dysphagia, and apneic periods due to contraction of the thoracic muscles and/or glottal or pharyngeal muscles. During a generalized spasm the patient arches his back, extends his legs, flexes his arms in abduction, and clenches his fists. Apnea results during some of the spasms. Autonomic overactivity initially manifests as irritability, restlessness, sweating, and tachycardia. Several days later this may present as hyperpyrexia, cardiac arrhythmias, labile hypertension, or hypotension. | 65 y/o m, cannot open mouth, contraction of muscles causing painful spasms for minutes, sweating, tachycardia, cut hand while gardening, did not get tetanus shot | “A 65-year-old man cannot open his mouth (lockjaw), and has painful spasms all over his body lasting several minutes. He is sweating and his heart is racing. Before his symptoms occurred, he cut his hand while gardening. He had not been vaccinated against tetanus”. |  | SCs: 50%  Subjects: 98.9% |
| **Non-Emergent** | | | | |  |
| 16 | An 18-month-old toddler presents with 1 week of rhinorrhea, cough, and congestion. Her parents report she is irritable, sleeping restlessly, and not eating well. Overnight she developed a fever. She attends day care and both parents smoke. On examination signs are found consistent with a viral respiratory infection including rhinorrhea and congestion. The toddler appears irritable and apprehensive and has a fever. Otoscopy reveals a bulging, erythematous tympanic membrane and absent landmarks. | 18 mo f, 1 week rhinorrhea, cough, congestion, irritable, lack of appetite, fever, in daycare | “An 18-month-old girl has a runny nose, a cough and a stuffy (“congested”) nose for one week. She is irritable, lacks appetite and has a fever. She attends day-care”. |  | SCs: 75%  Subjects: 67.0% |
| 17 | A 7-year-old girl presents with abrupt onset of fever, nausea, vomiting, and sore throat. The child denies cough, rhinorrhea, or nasal congestion. On physical exam, oral temperature is 101°F (38.5°C) and there is an exudative pharyngitis, with enlarged cervical lymph nodes. A rapid antigen test is positive for group A Streptococcus (GAS). | 7 y/o f, fever (101), nausea, vomiting, sore throat, swollen lymph nodes, tonsilar exudate; no cough, rhinorrhea, or nasal congestion | A 7-year-old girl suddenly developed a fever (101° Fahrenheit; 38.3° Celsius), with nausea, vomiting and a sore throat. The lymph nodes on her neck are swollen and her tonsils are covered with white spots (“tonsillar exudate”). She does not have a cough, and her nose is not runny and clear (i.e. not congested)”. |  | SCs: 53.8%  Subjects: 68.1% |
| 18 | Mr. A is a 24 year-old man who presents to your office for complaints of sore throat, fever, and headache. His symptoms started 2 days ago with acute onset of sore throat and fever to 102.2. He has had no cough. His physical examination is normal, except for the presence of tonsillar exudates and some tender anterior cervical lymphadenopathy. He is otherwise in good health, and is on no medications except for ibuprofen for fever. He has no drug allergies. (, Centor score = 4 – treat, or test and treat) | 24 y/o m, sore throat, fever (102.2), headache, no cough,tonsilar exudates | A 24-year-old man has a sore throat and a fever of 102.2° Fahrenheit (39° Celsius). He also complains about a headache. He has no cough. His tonsils are covered with white spots (“tonsillar exudate”)”. |  | SCs: 84.6%  Subjects: 75.8% |
| 19 | Mrs. S is a 35 year-old woman who presents with 15 days of nasal congestion. She has had facial pain and green nasal discharge for the last 12 days. She has had no fever. On physical examination, she has no fever and the only abnormal finding is maxillary tenderness on palpation. She is otherwise healthy, except for mild obesity. She is on no medications, except for an over-the-counter decongestant. She has no drug allergies | 35 y/o f, sx for 15 days, nasal congestion, facial pain, green nasal discharge, no fever | A 35-year old woman has had a stuffy nose for 15 days. She also complains of facial pain. Her nasal discharge is green. She has no fever”. |  | SCs: 50%  Subjects: 72.5% |
| 20 | Consider a 35-year-old man who developed low back pain after shoveling snow 3 weeks ago. He presents to the office for an evaluation. On examination there is a new left foot drop. In study 82% physicians recommend MRI (sciatica/sprain) | 35 y/o m, back pain following shoveling, left foot drop, symptoms 3 weeks of duration (loss of sensation in foot) | “A 35-year-old man developed lower back pain after shoveling snow. It is difficult for him to lift the front part of the left foot. Also, he lost sensation in his left foot”. The symptoms have been present for three weeks already. | Inherited Semigran’s addition of “loss of sensation in foot”, though not present in original version. | SCs: 33.3%  Subjects: 73.6% |
| 21 | A 45-year-old man presents with acute onset of pain and redness of the skin of his lower extremity. Low-grade fever is present and the pretibial area is erythematous, edematous, and tender. | 45 y/o m, pain and redness of skin, low grade fever, redness, edema, and tenderness lower leg | “A 45-year old man suddenly develops pain and redness of the skin on his lower leg. He also has a low-grade fever. The area over his shin is red, swollen and painful to the touch”. |  | SCs: 22.2%  Subjects: 67.0% |
| 22 | A 56-year-old woman with a history of smoking presents to her primary care physician with shortness of breath and cough for several days. Her symptoms began 3 days ago with rhinorrhea. She reports a chronic morning cough productive of white sputum, which has increased over the past 2 days. She has had similar episodes each winter for the past 4 years. She has smoked 1 to 2 packs of cigarettes per day for 40 years and continues to smoke. She denies hemoptysis, chills, or weight loss and has not received any relief from over-the-counter cough preparations. | 56 y/o f, Hx of smoking, shortness of breath and cough for several days, rhinorrhea 3 days ago, white sputum, no chills | “A 56-year-old woman, who is a heavy smoker, complains of shortness of breath and a cough, both of which have already lasted several days. It started out 3 days ago with a runny nose. She is coughing up white mucus and does not have chills (i.e. is not shivering). |  | SCs: 23.0%  Subjects: 74.7% |
| 23 | A 30-year-old woman presents in January with 2-day history of fever, cough, headache, and generalized weakness. She was in her usual state of health before an abrupt onset of these symptoms. A few viral illnesses have affected her during the current winter, but not to this severity. She reports sick contacts at work and did not receive the seasonal influenza vaccine this season. | 30 y/o f, 2 day fever, cough, headache, weakness, did not get flu shot | “A 30-year-old woman has had fever for two days. Now, she also complains of a cough, headache, and weakness. She did not get this season’s flu shot.” |  | SCs: 33.3%  Subjects: 47.2% |
| 24 | A 16-year-old female high school student presents with complaints of fever, sore throat, and fatigue. She started feeling sick 1 week ago. Her symptoms are gradually getting worse, and she has difficulty swallowing. She has had a fever every day, and she could hardly get out of bed this morning. She does not remember being exposed to anybody with a similar illness recently. On physical examination she is febrile and looks sick. Enlarged cervical lymph nodes, exudative pharyngitis with soft palate petechiae and faint erythematous macular rash on the trunk and arms are found. | 16 y/o f, 1 week Hx of fever, sore throat, fatigue, difficulty swallowing, fever, enlarged lymph nodes, exudates, macular rash on trunk/arms | “A 16-year-old girl has had a fever for one week, accompanied by a sore throat, fatigue, difficulty swallowing, and enlarged lymph nodes on her neck. The back of her throat is covered with white spots. She has flat rashes on her arms and torso.” |  | SCs: 75%  Subjects: 65.9% |
| 25 | A 40-year-old man presents to his primary care physician with a 2-month history of intermittent upper abdominal pain. He describes the pain as a dull, gnawing ache. The pain sometimes wakes him at night, is relieved by food and drinking milk, and is helped partially by ranitidine. He had a similar but milder episode about 5 years ago, which was treated with omeprazole. Physical examination reveals a fit, apparently healthy man in no distress. The only abnormal finding is mild epigastric tenderness on palpation of the abdomen. | 40 y/o m, 2 month Hx of intermittent upper abdominal pain, dulling and gnawing ache, wakes at night and is relieved by food/drinking milk/ranitidine, prior episode 5 yrs ago | A 40 year-old-man suffers from periodic upper stomach pain, which he has had for the past two months. He describes the pain as a dull and gnawing ache. The pain sometimes wakes him up at night, and can be relieved by eating, drinking milk or taking **ranitidine** (a medication reducing the stomach acid production, hence fighting heart burn). He had a similar episode about five years ago”. | Explained ranitidine | SCs: 66.7%  Subjects: 74.7% |
| 26 | A 6-year-old boy with a medical history significant for mild persistent asthma is brought to the clinic by his mother with a history of a 5-day cough. His mother reports that the child's fever continues to be elevated despite acetaminophen therapy. He has missed school for the past 3 days and he has a classmate sick with pneumonia. The mother reports that the appetite is good for the child. His cough produced yellowish sputum at home. His vitals at the clinic are: respiratory rate 19 breaths/min, heart rate 80 beats/min, and temperature 101.6°F (38.7°C). He appears in no respiratory distress. His lung examination reveals bilateral rales and occasional wheeze. CXR reveals lobar infiltrates without pleural effusions. | 6 y/o m, Hx of asthma, 5 days cough, fever, appetite good, yellow sputum, t 101.6 | “A 6-year-old boy with asthma has had a cough for the last five days. He also suffers from a fever. His appetite is good. He coughs up yellow mucus. His temperature is 101.6° Fahrenheit (38.7° Celsius)”. |  | SCs: 50%  Subjects: 79.1% |
| 27 | A 14-year-old boy presents with nausea, vomiting, and diarrhea. Eighteen hours earlier, he had been at a picnic where he ingested undercooked chicken along with a variety of other foods. He reports moderate-volume, nonbloody stools occurring 6 times a day. He has mild abdominal cramps and a low-grade fever. He is evaluated at an acute care clinic and found to be mildly tachycardic (heart rate 105 bpm) with a normal BP and a low-grade temperature of 100.1°F (37.8°C). His physical exam is unremarkable except for mild diffuse abdominal tenderness and mild increased bowel sounds. He is able to take oral fluids and is instructed on the appropriate oral fluid and electrolyte rehydration. | 14 y/o m, nausea, vomiting, non-bloody diarrhea, mild abdominal cramps (T=100.1), mild abdominal tenderness, diarrhea after attending a picnic and eating undercooked chicken, | “A 14-year-old boy suffers from nausea, vomiting, non-bloody diarrhea, and mild stomach cramps. He has a fever of 100.1° Fahrenheit (37.8° Celsius). His abdomen is painful to the touch. The diarrhea began after eating undercooked chicken at a picnic.” |  | SCs: 50%  Subjects: 39.5% |
| 28 | A 77-year-old man reports a 5-day history of burning and aching pain on the right side of his chest. This is followed by the development of erythema and a maculopapular rash in this painful area, accompanied by headache and malaise. The rash progressed to develop clusters of clear vesicles for 3 to 5 days, evolving through stages of pustulation, ulceration, and crusting. | 77 y/o m, 5 day burning and aching on right side of chest, erythema, maculopapular rash, headache, malaise, rash progressed to clear vesicles after 3-5 days | “A 77-year-old man complains of a burning aching sensation on the right side of his chest for the last five days. Initially, this area was red but developed into a rash. He feels ill and has a headache. The rash developed further into small blisters filled with clear fluid, which appeared after 3-5 days.” |  | SCs: 46.1%  Subjects: 49.4% |
| 29 | A 26-year-old female newly wed presents complaining of painful urination, feeling of urgent need to urinate, and more frequent urination for 2 days. She denies any fever, chills, nausea, vomiting, back pain, vaginal discharge, or vaginal pruritus. | 26 y/o f, painful urination, urgent need to urinate, more frequent urination for 2 days, sexually active; no fever, chills, nausea, vomiting, back pain, vaginal discharge, vaginal pruritus | “A 26-year old woman suffers from painful urination, constantly feels the need to go to the bathroom, and urinated more frequently in the last two days. She is sexually active, and does not have fever, chills (i.e. is not shivering), nausea, vomiting, back pain, vaginal discharge or vaginal itching”. |  | SCs: 72.7%  Subjects: 84.6% |
| 30 | A 65-year-old woman presents with a chief complaint of dizziness. She describes it as a sudden and severe spinning sensation precipitated by rolling over in bed onto her right side. Symptoms typically last <30 seconds. They have occurred nightly over the last month and occasionally during the day when she tilts her head back to look upward. She describes no precipitating event prior to onset and no associated hearing loss, tinnitus, or other neurologic symptoms. Otologic and neurologic examinations are normal except for the Dix-Hallpike maneuver, which is negative on the left but strongly positive on the right side. | 65 y/o f, dizziness, sudden onset, recurrent, lasts <30 sec, consistent trigger, no hearing loss, ringing in ears, muscle weakness, loss of sensation | “A 65-year-old woman complains of dizziness (severe spinning sensation). She describes the dizziness as appearing suddenly. The dizziness lasts less than 30 seconds and is consistently triggered by tilting her head back. She has no hearing loss, no ringing in the ears, no muscle weakness or loss of sensation”. | Consistent trigger specified as in the original version (“tilting her head back”) | SCs: 88.8%  Subjects: 86.8% |
| **Self-Care** | | | | |  |
| 31 | A 34-year-old woman with no known underlying lung disease 12-day history of cough. She initially had nasal congestion and a mild sore throat, but now her symptoms are all related to a productive cough without paroxysms. She denies any sick contacts. On physical examination she is not in respiratory distress and is afebrile with normal vital signs. No signs of URI are noted. Scattered wheezes are present diffusely on lung auscultation. | 34 y/o f, 12 day cough, initial nasal congestion and sore throat, cough, no fever | “A 34-year-old woman has been suffering from a cough for 12 days. Initially, she had a stuffy nose and a sore throat. She has no fever”. |  | SCs: 61.5%  Subjects: 59.3% |
| 32 | Mrs. L is a 61 year-old woman who presents with 4 days of a cough productive of yellow sputum. Her symptoms started 4 days ago with rhinorrhea and productive cough. She initially had fevers as high as 101 for 2 days, but those have now resolved. In the office, she has normal vital signs and a normal physical examination. She is otherwise healthy except for high cholesterol for which she is being treated with atorvastatin. She has no drug allergies. | 61 y/o f, 4 day cough, yellow sputum, rhinorrhea, fever (resolved) | “A 61-year-old woman has been suffering from cough for four days. She is still coughing up yellow mucus. Also, she has a runny nose. An initial fever has now gone away”. |  | SCs: 41.6%  Subjects: 76.9% |
| 33 | A 14-year-old boy with no significant past medical history presents 3 days after developing a red, irritated right eye that spread to the left eye today. He has watery discharge from both eyes and they are stuck shut in the morning. He reports recent upper respiratory symptoms and that several children at his day camp recently had pink eye. He denies significant pain or light sensitivity and does not wear contact lenses. On examination, his pupils are equal and reactive and he has a right-sided, tender preauricular lymph node. Penlight examination does not reveal any corneal opacity. | 14 y/o m, 3 days red, irritated eye (spread from right to left), discharge, URI symptoms, no pain or light sensitivity | “A 14-year-old boy has had a red, irritated right eye for three days, and as of today, his left eye is also affected. Both his eyes produce a watery discharge. Recently he had symptoms of an upper airway infection, but he has no pain and is not over-sensitive to light”. |  | SCs: 16.6%  Subjects: 4.7% |
| 34 | Mr. E is a 26 year-old man who presents to your office for complaints of sore throat, headache, and non-productive cough. His symptoms started 2 days ago with acute onset of sore throat. He has been afebrile. His physical examination is normal, except for some pharyngeal erythema. He is otherwise in good health, and is on no medications except for acetaminophen for his sore throat and fever. He has no drug allergies. | 26 y/o m, 2 day sore throat, headache, cough, no fever | “A 26-year-old man has been suffering from a sore throat for two days. He also has a headache and a cough but no fever”. |  | SCs: 61.5%  Subjects: 89.0% |
| 35 | A 22-year-old student presents with a 5-year history of worsening nasal congestion, sneezing, and nasal itching. Symptoms are year-round but worse during the spring season. On further questioning it is revealed that he has significant eye itching, redness, and tearing as well as palate and throat itching during the spring season. He remembers that his mother told him at some point that he used to have eczema in infancy. | 22 y/o m, 5 year Hx of nasal congestion, sneezing, nasal itching worse during spring season, eye itching, redness, tearing, palate and throat itching, Hx of eczema in infancy | “A 22-year-old man complains of a stuffy nose, nasal itching, and sneezing that have been getting worse during the last five years. His symptoms are worse during the spring season. Currently, his eyes are red and itchy, with tearing. His palate and throat also itch. In his infancy he suffered from eczema”. |  | SCs: 45.4%  Subjects: 47.2% |
| 36 | A 38-year-old man with no significant history of back pain developed acute LBP when lifting boxes 2 weeks ago. The pain is aching in nature, located in the left lumbar area, and associated with spasms. He describes previous similar episodes several years ago, which resolved without seeing a doctor. He denies any leg pain or weakness. He also denies fevers, chills, weight loss, and recent infections. Over-the-counter ibuprofen has helped somewhat, but he has taken it only twice a day for the past 3 days because he does not want to become dependent on painkillers. On examination, there is decreased lumbar flexion and extension secondary to pain, but a neurologic exam is unremarkable. | 38 y/o m, acute low back pain after lifting, no leg pain or weakness, no fevers, chills, weight loss, or recent infections | “A 38-year-old man developed lower back pain suddenly after lifting boxes. He has no pain or weakness in his legs, and he had no fever, no chills (i.e. is not shivering), no weight loss nor a recent infection”. | Stuck with Semigran version despite it missing the temporality “two weeks ago” | SCs: 33.3%  Subjects: 76.9% |
| 37 | A 9-year-old boy is brought to the ER after being stung by a bee at a picnic. He is crying hysterically. After 15 minutes of calming him down, exam reveals a swollen tender upper lip but no tongue swelling, no drooling, no stridor, no rash, and no other complaints. | 9 y/o m, bee sting, swollen and tender upper lip; no tongue swelling, drooling, stridor, rash, or other complaints | “A 9-year-old boy was stung by a bee. His upper lip is swollen and is painful to the touch. The tongue is not swollen, and he is not drooling or having difficulty breathing (i.e. no “stridor”). There is no rash or any other complaints”. |  | SCs: 11.1%  Subjects: 87.9% |
| 38 | A 17-year-old male student presents with recurrent mouth ulceration since his early schooldays. He has no respiratory, anogenital, gastrointestinal, eye, or skin lesions. His mother had a similar history as a teenager. The social history includes no tobacco use and virtually no alcohol consumption. He has no history of recent drug or medication ingestion. Extraoral exam reveals no significant abnormalities and specifically no pyrexia; no cervical lymph node enlargement; nor cranial nerve, salivary, or temporomandibular joint abnormalities. Oral exam reveals a well-restored dentition and there is no clinical evidence of periodontal-attachment loss or pocketing. He has five 4 mm round ulcers with inflammatory haloes in his buccal mucosae. | 17 y/o m with recurrent mouth ulceration for year, no respiratory, anogenital, gastrointestinal, eye, or skin lesions, mother has similar Hx, no Hx of recent drugs or medication | “A 17-year-old boy complains of having mouth sores again and again since childhood. He has no such lesion in other any other areas. His mother had similar complaints as a teenager. He does not smoke, use any drugs, or take any medication”. | Omitted “no respiratory, gastrointestinal, anogenital or eye lesion” for brevity | SCs: 27.2%  Subjects: 29.6% |
| 39 | Consider a 40-year-old, monogamous, married woman who calls to report a 2-day history of vaginal itching and thick white discharge. She has no abdominal pain or fever. (in study 50% recommended physician visit) | 40 y/o f, 2 day vaginal itching, thick white discharge, no abdominal pain or fever | “A 40-year-old woman has vaginal itching and thick white vaginal discharge for 2 days. She does not have stomach pain or fever”. |  | SCs: 11.1%  Subjects: 34.0% |
| 40 | A 5-month-old baby boy presents with difficulty and delay in passing hard stools. His mother reports that he strains for several hours and may even miss a day, before passing stool with screaming and occasional spots of fresh blood on the stool or diaper. He has recently been weaned from breastfeeding to cows' milk formula, which he had been reluctant to drink initially. The child is thriving and now feeding normally. There was no neonatal delay in defecation and no history of excessive vomiting or abdominal distension. | 5 mo m, difficulty/delay in passing hard stools, strains for hours, may miss a day, screams when passes stool and occasional spots of blood, weaned from breastmilk to cows' milk, now feeding normally | “A 5-month-old baby boy experiences difficulties and delay in passing hard stools. His mother reports that he strains for several hours and may even miss a day before passing stool with screaming and occasional spots of blood on the stool or diaper. He has recently been weaned from breast to cow’s milk and is now feeding normally”. |  | SCs: 9.1%  Subjects: 9.8% |
| 41 | A 12-year-old female presents with dry, itchy skin that involves the flexures in front of her elbows, behind her knees, and in front of her ankles. Her cheeks also have patches of dry, scaly skin. She has symptoms of hay fever and has recently been diagnosed with egg and milk allergy. She has a brother with asthma and an uncle and several cousins who have been diagnosed with eczema. | 12 y/o f, dry, itchy skin in front of elbows, behind knees, in front of ankles, cheeks have patches of dry, scaly skin, symptoms of hay fever, egg and milk allergy, brother has asthma and uncle and cousins have eczema | “A 12-year-old girl has dry and itchy skin on the front of her elbows, behind the knees, and in front of her ankles. Her cheeks have patches of dry and scaly skin. She also has symptoms of hay fever and has an egg and milk allergy. Her brother has asthma, and her uncle and cousins have eczema”. |  | SCs: 25%  Subjects: 21.9% |
| 42 | A 30-year-old man presents with a painful, swollen right eye for the past day. He reports minor pain on palpation of the eyelid and denies any history of trauma, crusting, or change in vision. He has no history of allergies or any eye conditions and denies the use of any new soaps, lotions, or creams. On exam, he has localized tenderness to palpation and erythema on the midline of the lower eyelid near the lid margin. The remainder of the physical exam, including the globe, is normal. | 30 y/o m, painful, swollen right eye for past day, no Hx of trauma, crusting, change in vision, allergies, or eye conditions, localized tenderness, erythema (redness) | A 30-year-old man complains of pain and swelling on his right eye for the past day. There was no previous trauma or crusting of the eye. He has no allergies, no change in vision, and no other eye-related conditions. His lower eyelid is reddened and painful to the touch. |  | SCs: 33.3%  Subjects: 27.4% |
| 43 | Mr. R. is a 56 year-old man who presents to you with 6 days of non-productive cough, nasal congestion, and green nasal discharge. He has had intermittent fevers as high as 100.8. His physical examination is normal except for rhinorrhea. He is otherwise healthy, except for chronic osteoarthritis of the right knee. He has no drug allergies. | 56 y/o m, 6 day cough, nasal congestion, green nasal discharge, fever (100.8), rhinorrhea | A 56-year-old man has had a cough for 6 days, a stuffy nose with green nasal discharge (mucus), and a fever of 100.8° Fahrenheit (38.2° Celsius). The nose is also runny. |  | SCs: 50%  Subjects: 42.8% |
| 44 | A30-year-old man presents with a 2-day history of runny nose and sore throat. He feels hot and sweaty, has a mild headache, is coughing up clear sputum and complains of muscle aches. He would like antibiotics as he was prescribed them last year when he had a similar condition. On examination, he is afebrile, has a normal pulse, a slightly inflamed pharynx and nontender cervical lymphadenopathy. There is no neck stiffness and his chest is clear. He has tried over-the-counter cough medications, but has not found these helpful. He smokes 10 cigarettes per day. | 30 y/o m, 2 day HX of runny nose, sore throat, hot, sweaty, mild headache, cough with clear sputum, muscle aches, no fever or neck stiffness | “A 30-year-old male complains of a runny nose and a sore throat which has been ongoing for 2 days. He feels hot and sweaty, has a mild headache, is coughing up clear mucus, and complains of muscle aches. He has no fever or neck stiffness”. |  | SCs: 58.3%  Subjects: 71.4% |
| 45 | Elizabeth’s 2-year-old son has a fever and vomited twice. Elizabeth worries about dehydration, so she gives Jack a sippy cup of apple juice. He immediately vomits up the juice. Elizabeth debates what to do next. Should she try to reach Jack’s pediatrician or should she take Jack to the ED? Instead, she calls her triage nurse line. Temperature = 100.5 | 2 y/o m, low grade fever (T = 100.5), vomited twice, vomits up juice | “A 2-year-old boy has a low fever of 100.5° Fahrenheit (38.1° Celsius) and has vomited twice. His mother tried giving him juice in a sippy cup, but he immediately vomited up the juice.” |  | SCs: 0%  Subjects: 20.8% |

1. See supplementary material of Semigran et al., 2015 for the list of sources. [↑](#endnote-ref-1)
2. See supplementary material of Semigran et al., 2015 [↑](#endnote-ref-2)
3. Case difficulty is defined as proportion of symptom checkers or subjects assessing the case vignette correctly of all SCs or subjects who evaluated this case vignette. [↑](#endnote-ref-3)
